# AI-driven proactive music therapy in the era of digital mental health

**DOI:** 10.3389/fpsyg.2026.1832950

**Published:** 2026-05-14

**Authors:** Feng Li, Chengmeng Zhang, Chang Liu, Hanyi Jia, Zaishuo Wang

**Affiliations:** 1 Anyang Normal University, Anyang, Henan, China; 2 Institute of Population Research, Peking University, Beijing, China; 3 Fryderyk Chopin University of Music, Warsaw, Poland; 4 Yiwu Art School, Yiwu, Zhejiang, China

**Keywords:** digital mental health, emotion recognition, music therapy, personalized music therapy, proactive intervention

## Abstract

Digital technologies such as telepsychology, mobile health applications, artificial intelligence (AI), and immersive virtual environments are rapidly transforming the delivery of psychological care. Despite these advances, music therapy remains weakly integrated into most digital mental health systems. In many current interventions, including virtual reality therapies and mental health applications, music is typically used as background ambience rather than as an active therapeutic mechanism. This disconnect limits the potential of music-based interventions for emotional regulation and psychological support. Advances in artificial intelligence create new opportunities to address this gap. Through emotion recognition, behavioral data analysis, and generative music algorithms, AI systems can anticipate emotional states and deliver adaptive musical interventions before psychological distress escalates. Such AI-driven proactive music therapy enables music to function as an embedded regulatory component within digital mental health ecosystems rather than as a passive environmental feature. A conceptual framework for integrating proactive music therapy into digital mental health platforms is proposed, highlighting key technological components including emotion sensing, adaptive music intelligence, and digital therapeutic delivery. Ethical considerations and research priorities for AI-enabled music interventions are also outlined. AI-driven proactive music therapy may represent an important direction for scalable and personalized psychological care in the era of digital mental health.

## 1 Introduction

Digital technologies are rapidly transforming the landscape of mental health care. Telepsychology platforms, mobile mental health applications, artificial intelligence (AI) systems, and immersive virtual environments are expanding the ways psychological assessment and intervention can be delivered. These innovations have significantly increased accessibility to mental health services and have created new opportunities for personalized and scalable psychological support. In particular, the emergence of digital therapeutics and technology-assisted therapy models has reshaped how clinicians conceptualize the delivery of psychological care.

At the same time, there has been renewed interest in integrating established therapeutic approaches into digital mental health systems. Among these approaches, music therapy has long been recognized as a powerful tool for emotional regulation and psychological support. Research across psychology, neuroscience, and behavioral medicine has shown that music can influence emotional processing, reduce anxiety, modulate stress responses, and support cognitive and psychological wellbeing (Blood and Zatorre, 2001; Aalbers et al., 2017; Erkkilä et al., 2011). Music-based interventions have been used in a variety of clinical contexts, including depression, anxiety disorders, trauma recovery, and neurological rehabilitation (Aalbers et al., 2017; Erkkilä et al., 2011; Picard, 1997).

Despite these well-documented benefits, music therapy remains surprisingly under-integrated within contemporary digital mental health technologies. In many digital therapeutic environments, including virtual reality therapies, mental health mobile applications, and online psychological interventions, music is typically used only as background ambience rather than as an active therapeutic mechanism. Even in immersive digital interventions that integrate sophisticated visual and behavioral components, music often plays a secondary role that does not fully reflect its potential as a therapeutic tool. Music is often implemented as a peripheral or ambient feature, despite its established therapeutic potential. So, when considered alongside advances in music therapy and affective computing, points to a structural gap between traditional music therapy practices and emerging digital psychological technologies (Torous et al., 2021; Choong, 2024). This gap may be further exacerbated by broader challenges in digital accessibility. Existing research indicates that older adults continue to face significant digital divides in accessing and benefiting from information technologies, particularly in the context of mental health and wellbeing interventions (Zhang et al., 2023). Similar challenges have also been reported among individuals with disabilities, low-resource populations, culturally diverse groups, and those with limited digital literacy, highlighting broader issues of accessibility and equity in digital mental health systems (Van Kessel et al., 2022). When digital systems are not designed to be adaptive, personalized, and inclusive, the therapeutic potential of modalities such as music becomes even less accessible to populations that could benefit the most. As a result, the under-integration of music therapy is not only a technological issue, but also a question of equitable access in digital mental health ecosystems.

Proactive music therapy refers to AI-enabled systems that anticipate emotional dysregulation and deliver adaptive musical interventions before psychological distress escalates. Artificial intelligence provides a unique opportunity to bridge this gap. Advances in affective computing, machine learning, and generative algorithms make it possible to develop adaptive music systems that respond dynamically to an individual's emotional and physiological states (Juslin and Västfjäll, 2008; Zeng et al., 2009; Calvo and D'mello, 2010). Recent studies also highlight the therapeutic value of active and technology-supported music interventions (Schneider et al., 2022; Jiao, 2025; Wei and He, 2026). Through real-time emotion recognition, personalized music recommendation, and AI-generated soundscapes, music can become an embedded therapeutic mechanism within digital mental health environments rather than merely a passive background element (Zeng et al., 2009; Shaffer and Ginsberg, 2017).

Building on this perspective, AI-driven proactive music therapy can be conceptualized as a new paradigm for digital mental health care. A clearer understanding of this paradigm requires examining the structural disconnect between music therapy and digital psychological technologies, as well as the role of artificial intelligence in enabling adaptive and proactive musical interventions. These developments also point to the need for a practical implementation pathway that integrates AI-driven music therapy into digital mental health systems, alongside careful consideration of ethical and clinical challenges.

## 2 State of the field: music therapy, affective computing, and digital mental health

Music therapy, affective computing, and digital mental health have each developed substantial bodies of research, yet their integration remains limited. Understanding the current state of these domains is essential for situating the proposed framework.

Within clinical psychology, music therapy has been widely applied as an intervention for emotional regulation, stress reduction, and mental health treatment. Music therapy and music-based intervention research have established a relatively robust effectiveness in the domains of affective disorders (like depression, anxiety disorders, and trauma-related symptoms) and neurological rehabilitation (Williams and Sidis, 2025; Aalbers et al., 2017; Lorek et al., 2023). At the same time, research on music-evoked emotion has provided an important theoretical basis for understanding its therapeutic potential, musical emotions may not arise through a single pathway, but rather through multiple mechanisms, including brain stem reflexes, emotional contagion, episodic memory, and musical expectancy (Sayal et al., 2025). Evidence suggests that music modulates brain networks associated with reward, emotion, and autonomic regulation (Koelsch, 2014). However, most music therapy interventions are delivered in therapist-led or semi-structured settings, with limited scalability and minimal integration into digital systems. Existing digital adaptations often rely on static playlists or user-selected music, which lack real-time responsiveness to changing emotional states.

In parallel, affective computing has advanced rapidly, enabling the detection and modeling of emotional states through multimodal data sources such as speech, facial expressions, and physiological signals (Wang et al., 2022). This technology provides the key technical preconditions for real-time sensing, dynamic adaptation, and closed-loop regulation in music-based intervention. Some studies suggest that emotion monitoring could be linked to the design of music interventions, and that both wearable and non-wearable sensing approaches point to the practical feasibility of a closed loop between emotion monitoring and music-based regulation (Vuijk et al., 2023; Guo et al., 2024). While machine learning and deep learning techniques have been applied to infer affective states and support adaptive human–computer interaction, providing the technical foundation and methodological basis for making music therapy more real-time and individualized, but their application in clinical music therapy remains underdeveloped, and few systems translate affective signals into structured therapeutic interventions.

Digital mental health technologies, including mobile health applications, telepsychology platforms, and virtual reality interventions, have expanded access to psychological care and enabled new forms of engagement. Recent meta-analytic evidence suggests that smartphone-based mental health applications can produce small but statistically significant improvements in depression and generalized anxiety symptoms (Linardon et al., 2024). Although the evidence and future directions of apps, social media, chatbots, and virtual reality within digital psychiatry was fully discussed by scholars, but within these systems, music is typically used as a background feature, often limited to enhancing atmosphere, rather than shaping therapeutic processes (Torous et al., 2025, 2021; Choong, 2024). As summarized in Table 1, representative studies across these domains reveal a fragmented landscape, with limited integration between emotional sensing, adaptive music generation, and clinically grounded intervention design.

**Table 1:** Representative studies across music therapy, affective computing, and digital mental health.

Research domain	Representative study	Methodology	Role of music	Key findings/contributions
Music therapy (clinical evidence and mechanisms)	Aalbers et al., 2017; Williams and Sidis, 2025	Systematic review	Therapeutic intervention	Supportive evidence that music therapy reduces depressive symptoms
	Erkkilä et al., 2011	Randomized controlled trial	Individual music therapy	Significant reduction in depressive symptoms compared to treatment as usual
	Sihvonen et al., 2017	Narrative review	Therapeutic intervention	Positive effects on motor, cognitive, and emotional outcomes
Affective computing	Calvo and D'mello, 2010	Interdisciplinary review	None	Comprehensive review of affect detection approaches and applications
	Wang et al., 2022	Systematic review	None	Comprehensive taxonomy of affective computing across unimodal approaches
	Vuijk et al., 2023	Systematic review	Proposed integration: emotion monitoring + adaptive music intervention	Identifies gap between emotion sensing and music intervention for people with dementia; advocates human-centered, privacy-aware pervasive systems
	Guo et al., 2024	Experimental study	Adaptive therapeutic intervention	Demonstrated feasibility of integrating real-time emotion sensing with personalized music therapy in a wearable app context
Digital mental health	Mohr et al., 2014	Perspective/viewpoint	None	Proposes integrated framework for effective digital interventions
	Torous et al., 2021	Review	None	Synthesis of current evidence and future directions in digital psychiatry
	Linardon et al., 2024	Systematic review	None	Apps demonstrate small-to-moderate effects for depression and anxiety symptoms
	Choong, 2024	Interpretivist case study	Active therapeutic medium via electronic music technology	Demonstrated that digital tools can help achieve therapeutic goals (e.g., emotional regulation, self-expression, engagement) in mental health contexts

Taken together, these domains reveal while music therapy provides a clinically validated intervention, affective computing offers real-time emotional sensing, and digital mental health platforms enable scalable delivery, these components are rarely integrated into a unified system. The proposed framework addresses this gap by integrating these components into an AI-driven, proactive, and embedded model of music therapy within digital mental health ecosystems.

## 3 The missing therapeutic layer: music therapy in digital mental health systems

Artificial intelligence has emerged as a transformative force across many areas of health care, including mental health assessment and intervention, and its implications for music therapy are particularly consequential (Sun et al., 2023). AI-based systems can now detect emotional states from voice patterns, facial expressions, physiological signals, and behavioral data, forming the foundation of affective computing (Thaut, 2013; Thaut and Hoemberg, 2025; Briot et al., 2017). These capacities matter specifically for music therapy because the mechanism of music's therapeutic action is inherently continuous and state-dependent: the same musical stimulus produces divergent psychophysiological responses depending on baseline arousal, attentional focus, and contextual framing (Konieczna-Nowak, 2015; Jeon et al., 2026). Static digital interventions cannot account for this variability, and it is precisely here that AI-mediated systems introduce a structurally different possibility (Korsakova-Kreyn and Fedotchev, 2026).

Three interrelated capabilities define this shift. First, emotion recognition technologies enable digital platforms to infer users' psychological states in real time, drawing on speech patterns, facial micro-expressions, and physiological indicators such as heart rate variability to provide continuous affective feedback (Thaut and Hoemberg, 2025; Bishop and Nasrabadi, 2006). Second, machine learning algorithms can translate these affective signals into dynamically adjusted musical parameters, including tempo, rhythm, melody, and harmonic structure (Sihvonen et al., 2017; Torous et al., 2020), aligning intervention features with moment-to-moment psychological states rather than approximating them through static playlist logic. Third, generative AI architectures extend this further by producing adaptive music compositions in real time, such that the musical output evolves continuously as the user's emotional state changes (Firth et al., 2017; Xiao, 2025). This progression from detection to adaptation to generation constitutes a closed-loop therapeutic model, a structural departure from the episodic, content-delivery logic of existing digital mental health tools.

Another dimension concerns temporal continuity across sessions, which neither affective computing nor generative models alone address. The therapeutic efficacy of music therapy in face-to-face contexts derives substantially from accumulated clinical responsiveness over time: the therapist learns what works, tracks longitudinal patterns, and adjusts the arc of intervention accordingly (Schneible et al., 2023; Lineweaver et al., 2022). Machine learning systems are well suited to this kind of modeling. Preference trajectories, response patterns, and symptom fluctuations can be represented as time-series data and used to refine intervention parameters across sessions (Freeman et al., 2018), approximating the individualized attunement that characterizes effective clinical practice without requiring continuous therapist involvement.

Together, these developments suggest that AI does not simply make music therapy more convenient to deliver digitally. Rather, it addresses the specific structural mechanisms by which music therapy has resisted digitization: its dependence on real-time responsiveness, individual calibration, and longitudinal continuity. The equation changes not because AI reduces the complexity of music therapy, but because it renders its clinically essential features computationally tractable for the first time.

## 4 Toward embedded AI-driven proactive music therapy

However, the shift toward embedded music therapy is not merely a matter of technological enhancement, but reflects a deeper reconceptualization of how therapeutic mechanisms are organized within digital mental health systems. In most existing interventions, therapeutic modalities are modular and discrete: cognitive tasks, behavioral prompts, and psychoeducational content are delivered as separate components, often requiring active user engagement. Music, when present, is typically treated as a peripheral element that accompanies these components without directly shaping their therapeutic logic (Shah et al., 2024). An embedded approach, by contrast, positions music as a continuous regulatory layer that operates alongside and within other therapeutic processes.

The integration of AI-driven music therapy into digital mental health systems requires a shift in how music is conceptualized within therapeutic technologies. Rather than viewing music therapy as a standalone intervention delivered only in specialized clinical contexts, it may be more productive to treat music as an embedded regulatory layer within digital therapeutic environments (Corrò et al., 2026). Rather than requiring explicit user interaction, adaptive music can modulate emotional states in the background, shaping attentional focus, physiological arousal, and affective tone in ways that influence how other interventions are experienced (Shen et al., 2025). For example, in a digital cognitive behavioral therapy module, dynamically adjusted music could facilitate emotional readiness, reduce cognitive resistance, and enhance engagement with therapeutic tasks. In immersive virtual environments, music can function as an affective scaffold that synchronizes with visual and interactive elements to produce coherent therapeutic experiences (Estrella-Juarez et al., 2023).

In this framework, music functions as a dynamic component of the digital therapeutic architecture. Anxiety, stress, and mood fluctuations often unfold over time and are influenced by subtle changes in internal states and environmental conditions (Ferguson et al., 2023). Interventions that rely solely on user-initiated engagement may fail to address these dynamics effectively (De Witte et al., 2022). By contrast, embedded music therapy enables a form of low-threshold, continuous intervention that adapts in real time, potentially supporting emotional stability without requiring sustained cognitive effort from users (Lai-Tan et al., 2023). Emotional and physiological data collected through sensors or user interactions can be analyzed by AI algorithms to identify psychological states (Gjoreski et al., 2016). Based on these signals, proactive music interventions can be generated or adapted in real time. The resulting music becomes part of an integrated therapeutic system designed to guide emotional regulation and support psychological wellbeing.

**Figure 1:**
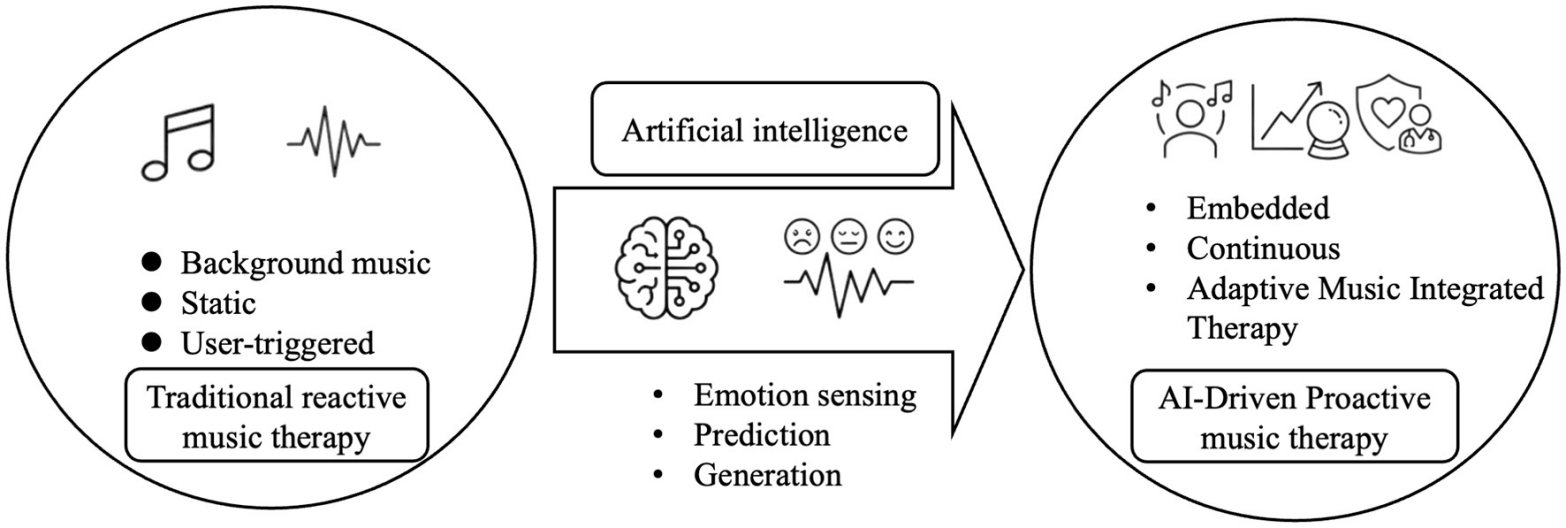
From reactive to proactive: AI-driven embedded music therapy in digital mental health.

Such embedded systems may operate across multiple digital platforms. Mobile mental health applications can deliver proactive music interventions throughout daily life, allowing users to regulate stress or mood outside clinical settings (Altenmüller and Schlaug, 2015; Koelsch, 2014). Telepsychology platforms can incorporate AI-driven music modules to complement therapist-guided sessions. Immersive virtual environments can integrate adaptive music into therapeutic simulations, enhancing emotional engagement and immersion (Insel, 2017; Zhao and Cheng, 2025). This conceptual shift from reactive and modular interventions to embedded, AI-driven proactive music therapy is illustrated in Figure 1.

As illustrated in Figure 1, this framework can be understood as a closed-loop system linking three core processes: emotion sensing, AI-based prediction and generation, and embedded therapeutic delivery. Emotional signals, captured through behavioral and physiological data, are continuously analyzed to infer user states. These signals are then translated by AI models into predictive and generative music responses, enabling adaptive intervention in real time. By embedding music therapy within digital mental health ecosystems, it becomes possible to combine the emotional power of music with the scalability and adaptability of artificial intelligence.

## 5 Implementation pathways

Developing AI-driven proactive music therapy systems will require coordinated progress across several technological and clinical domains. While the conceptual architecture of such systems is increasingly well-defined, translating it into deployable clinical tools demands attention to implementation specifics that are often underdressed in theoretical accounts.

The first component involves emotion sensing and data collection. Wearable devices, smartphones, and digital interaction platforms can provide continuous streams of physiological and behavioral data, including heart rate variability, electrodermal activity, speech patterns, and user interaction metrics, all of which serve as proxies for underlying emotional states (Bishop and Nasrabadi, 2006; Mohr et al., 2014; Vuijk et al., 2023). However, reliable affective inference in naturalistic settings remains technically demanding. Sensor noise, individual differences in physiological baseline, and context-dependent signal variability require robust preprocessing pipelines and adaptive calibration procedures. Importantly, data collection protocols must be designed with clinical populations in mind, as individuals experiencing anxiety, depression, or trauma-related conditions may respond differently to monitoring technologies, and perceived intrusiveness could itself undermine therapeutic engagement.

The second component involves AI-based music intelligence. Machine learning models must be developed to map emotional states to musical features that promote psychological regulation, drawing on established principles from neurologic music therapy and psychoacoustics to ensure that algorithmic outputs are clinically grounded rather than merely statistically optimized (Sihvonen et al., 2017; Hollis et al., 2017; Nunes et al., 2024). These models may incorporate both recommendation algorithms and generative music systems capable of creating adaptive sound environments in real time (Modran et al., 2023). A key implementation challenge here is validation: models trained on general population data may not generalize to specific clinical groups, and iterative refinement in collaboration with trained music therapists will be necessary to ensure that automated musical responses align with therapeutic intent rather than user preference alone.

The third component involves digital delivery platforms. Music-based interventions must be integrated into mobile applications, telepsychology platforms, and immersive environments where users can engage with therapeutic content in ecologically valid, real-world contexts. Integration with existing clinical infrastructure is particularly important. Standalone applications risk low long-term adherence if they operate independently of the broader therapeutic relationship; embedding music therapy modules within established telepsychology platforms, and enabling treating clinicians to monitor engagement data and adjust parameters, is more likely to support sustained use. Accessibility considerations, including platform usability across age groups, digital literacy levels, and device availability, must also inform design decisions from the outset rather than being treated as secondary concerns.

Finally, clinical monitoring and evaluation are essential. AI-driven music interventions should be evaluated through rigorous research methods, including randomized controlled trials and longitudinal studies, to determine their effectiveness across different populations and psychological conditions (Huckvale et al., 2020; Tarr et al., 2014). Outcome frameworks should extend beyond symptom reduction to include process measures such as therapeutic alliance, user engagement, and adherence trajectories (Schäfer et al., 2013; Yu et al., 2025), given that these factors are likely to mediate clinical outcomes in digital intervention contexts. Establishing minimum reporting standards for AI-driven music therapy research, analogous to those developed for other digital mental health interventions, would facilitate cumulative evidence synthesis and support eventual integration into evidence-based clinical guidelines.

## 6 Ethical and clinical considerations

As with many digital mental health technologies, the development of AI-driven music therapy raises important ethical and practical questions. One key concern involves data privacy. Emotion recognition systems often rely on sensitive personal data, including physiological signals and behavioral patterns (Sano and Picard, 2013; Gahlan and Sethia, 2025). Protecting the confidentiality and security of these data is essential for maintaining trust in digital therapeutic systems.

Another challenge involves algorithmic transparency and clinical validation. AI systems used in therapeutic contexts must be carefully evaluated to ensure that their recommendations are safe and clinically appropriate (Jiao, 2025). Interdisciplinary collaboration between clinicians, music therapists, engineers, and ethicists will be necessary to establish robust standards for the development and deployment of such systems (Torous et al., 2021; Dimaio and Engen, 2020).

Beyond technical feasibility, several clinical implementation barriers should be considered. Integrating AI-driven music therapy into existing clinical workflows may require alignment with therapist-led treatment structures and digital health platforms. Clinician acceptance is another important factor, as therapists may be cautious about delegating aspects of emotional regulation to automated systems. In addition, rigorous validation is necessary to ensure clinical efficacy and safety, particularly for vulnerable populations. Patient safety considerations, including the potential for unintended emotional responses to adaptive music, must also be addressed through careful system design and monitoring.

In addition, issues of accessibility and equity must be addressed. While digital technologies have the potential to expand access to mental health care (Dagum, 2018), disparities in digital literacy and technological infrastructure may limit their benefits for certain populations. Designing inclusive and accessible digital therapeutic systems should therefore be a priority.

To address these ethical concerns, more concrete governance approaches are required. Data governance frameworks should define how sensitive emotional and physiological data are collected, stored, and used, ensuring compliance with relevant regulations such as GDPR or HIPAA where applicable (Barbaria et al., 2025; Jonnagaddala and Wong, 2025). Transparent consent mechanisms are also necessary, allowing users to understand and control how their data inform adaptive interventions. In addition, system design should incorporate privacy-preserving techniques, such as on-device processing or data minimization. Aligning AI-driven music therapy systems with existing regulatory and clinical guidelines will be essential for responsible deployment.

## 7 Conclusion

The rapid expansion of digital mental health technologies presents new opportunities to rethink how therapeutic modalities are integrated into psychological care. Although music therapy has long been recognized as a powerful tool for emotional regulation, its potential has not yet been fully realized within digital therapeutic systems. In many current interventions, music remains a passive background element rather than an active therapeutic mechanism.

Artificial intelligence offers a pathway to change this situation. By enabling real-time emotion recognition, proactive music recommendation, and adaptive music generation, AI technologies make it possible to transform music into a dynamic regulatory component of digital mental health environments. AI-driven proactive music therapy therefore represents a promising direction for the next generation of digital mental health interventions.

Realizing this vision will require interdisciplinary collaboration, rigorous clinical research, and careful attention to ethical considerations. If these challenges can be addressed, the integration of AI-driven music therapy into digital mental health ecosystems may significantly expand the possibilities for scalable, personalized, and emotionally responsive psychological care.

## Data Availability

The original contributions presented in the study are included in the article/supplementary material, further inquiries can be directed to the corresponding author.
